# Dengue Fever in a Sickle Cell-Positive Pregnant Woman: A Case Report

**DOI:** 10.7759/cureus.67966

**Published:** 2024-08-27

**Authors:** Lucky Srivani Reddy, Manjusha Agrawal, Arpita Jaiswal, Deepika Dewani, Rishabh Dhabalia, Shaikh Muneeba

**Affiliations:** 1 Obstetrics and Gynecology, Jawaharlal Nehru Medical College, Datta Meghe Institute of Higher Education and Research, Wardha, IND; 2 Radiodiagnosis, Jawaharlal Nehru Medical College, Datta Meghe Institute of Higher Education and Research, Wardha, IND

**Keywords:** sickle cell crisis, treatment strategies, haematological disorders, immunity, complications, dual diagnosis, health challenges, sickle cell disease, dengue fever

## Abstract

Cases of sickle cell disease with dengue during pregnancy have rarely been reported. Sickle cell disorder is one of the most commonly inherited genetic disorders, especially in certain regions of India. Sickle cell disease, especially in pregnancy, has varying clinical severity, which may potentially lead to serious complications, negatively affecting the maternal and fetal outcomes. Dengue is commonly seen in tropical countries. Serotypes 1, 2, 3, and 4 of the dengue virus cause dengue, an infection spread by Aedes aegypti mosquitos. A 24-year-old primigravida with 36 weeks of gestation, with a known case of sickle cell disease and a history of multiple blood transfusions, presented to the emergency department with a history of fever for four days associated with body pains and chills. Her fever profile was sent, and the patient was diagnosed with dengue. She was treated with packed red cell transfusion and conservatively managed. She went into spontaneous preterm labour and delivered a healthy female child. Pregnancy-related pathophysiological changes, such as elevated blood volume, elevated metabolic demand, elevated blood viscosity, and hypercoagulability, combined with dengue fever complications, cause sickle cell disease patients to experience a higher rate of morbidity and mortality.

## Introduction

Among India's many tribal communities, the prevalence of sickle cell carriers ranges from 1 to 40% [1]. Pregnancy was linked to a high rate of maternal and foetal morbidity and mortality in the past among patients with sickle cell anaemia (SCA). Modern screening methods, maternal screening, and prophylactic vaccinations and antibiotics have all contributed to improvements in patient survival and overall disease outcomes, as well as a notable decline in the rates of maternal and foetal death [2]. All of the advancement accomplished hasn't changed the fact that pregnant sickle cell disease (SCD) women who contract dengue still have greater rates of clinical and obstetric problems than pregnant women in general. Pregnancy-related physiological changes in the circulatory, haematologic, renal, and pulmonary systems can put undue strain on organs already suffering from chronic injuries related to SCD. This may result in an elevated risk of acute chest syndromes, increasing vaso-occlusive crisis, anaemia, and preeclampsia, among other obstetric consequences. The increased risk of spontaneous abortions and stillbirths in these mothers is caused by microvascular injury and reduced uteroplacental circulation [3].

In the tropical and subtropical zones, dengue fever poses a threat to public health [4]. In recent decades, its prevalence has sharply increased worldwide [5]. Of the 390 million dengue cases reported each year, 96 million are symptomatic [6]. Additionally, data suggests that pregnant women are more likely to develop severe dengue illness than non-pregnant women [7]. Dengue shock syndrome (DSS) is a frequent consequence of dengue infection in pregnancy, and in cases with SCD, this fatality rate triples [8].

Pregnant women with SCD are thought to be at risk of developing severe dengue fever, according to World Health Organization (WHO) recommendations on the disease [9].

## Case presentation

A 24-year-old primigravida with 36 weeks of gestation, a booked case with regular antenatal visits which were uneventful. Known case of SCD (heterozygous type SS) with a history of multiple blood transfusions since childhood, presented to the emergency department with a history of fever for four days. It is associated with retro-orbital pain, epigastric pain, generalized body pain, and fever associated with chills. She had regular antenatal visits throughout her pregnancy, and no abnormality was detected.

Clinical examination

She had a fever of 102 degrees Fahrenheit, a blood pressure of 110/80 mmHg, a pulse rate of 110 beats per minute, a respiratory rate of 24 beats per minute, and a oxygen saturation (SpO2) of 99% on room air at admission. She appeared to be quite dehydrated. She had normal breath sounds. She had petechiae across her face, forearms, and both pretibial regions, measuring 1-2 mm in diameter.

On per abdominal examination, it was noted that her liver and spleen were mildly enlarged, and tenderness was observed. Her fundal height was around 36 weeks of gestation, and her uterus was relaxed, with a foetal heart rate of 164 beats/min. On per vaginal examination, her cervix was soft, anterior, 50% effaced, 2 cm dilated, and her station was -2 with no discharge seen (Bishop score 7). The patient's joints were examined to rule out sickle cell crisis; all the joints of the upper and lower limbs and the hip joint were examined, and no signs of joint tenderness were elicited. She had vague, generalised body pains.

Investigations

A complete blood count, a liver function test, a renal function test, a urine routine and microscopy, a urine culture, a high vaginal swab, a coagulation profile, a chest X-ray, and the patient's fever profile were among the blood studies that were done.

Laboratory analysis on admission gave the following results: haemoglobin 8.3 gm%, haematocrit (HCT) 35%, total leucocyte count 7,000/cumm; platelet count was 70,000/cumm, she tested positive for dengue NS1 antigen and immunoglobulin M (IgM), total bilirubin was 1.4mg/dL, lactate dehydrogenase (LDH) was 300U/L, the urine culture and high vaginal swab had no growth of bacteria and the chest X-ray was clear (Table 1).

**Table 1:** Lab values of the blood investigations done LDH- Lactate dehydrogenase

Blood investigations	Patients values	Normal values
Haemoglobin (gm%)	8.3	12-15
Haematocrit	35	36-48
Total leucocyte count (/cumm)	7,000	4,000-10,000
Platelet count (L/cumm)	70,000	1,50,000-3,50,000
Total bilirubin (mg/dL)	1.4	0.2-1.3
LDH (U/L)	300	150-300

Treatment

The patient was shifted to a dependency unit. She was immediately started on intravenous fluid replacement therapy to hydrate her along with intravenous acetaminophen 1000mg twice a day was started and continued till the fever completely subsided. She was closely monitored; her vitals were stable and showed no deterioration signs.

After 24 hours, the vital values were within normal ranges, and the epigastric pain had subsided. The haemoglobin was 8.3 gm%, HCT was 30%, and the platelet count was 60,000/mm3. Transfusion of one unit packed red cells was done under strict monitoring. The patient had a spontaneous rupture of membranes, due to which the patient went into preterm labour. The labour was actively managed. The patient was positioned in the left recumbent propped-up position and started on 2 litres of oxygen prophylactically throughout the labour. A 2.4-kilogram female child was born, who cried immediately after birth. Around 350 ml of blood was lost during labour. The baby's APGAR (appearance, pulse, grimace, activity and respiration) scores were 8 and 9 at one and five minutes of life. The baby was attended to by a neonatologist. No abnormality was detected in the newborn. The patient was monitored continuously for 24 hours, and her bleeding per vaginum was checked every two hours. Platelet concentrate was kept ready but not transfused as there was no severe bleeding.

Evolution

On the third day of admission, the patient gradually recovered. The haemoglobin was 9.2 gm%, and HCT was 31% with a platelet count of 85,000/cumm. She was discharged on the seventh day when her haemoglobin was 9.2 gm%; HCT was 31% with a platelet count of 1,00,000/cumm with no fever spikes, signs of sickle cell crisis and after contraceptive counselling. On one week follow-up, she was healthy with no episodes of fever, well-involuted uterus and haemoglobin of 9.2 gm%; HCT was 32% with a platelet count of 1,74,000/cumm.

## Discussion

The dengue virus, which is spread by the Aedes aegypti mosquito, has four serotypes: 1, 2, 3, and 4. It is a significant issue for public health in tropical nations. Dengue hemorrhagic fever (DHF) was divided into four grades based on its severity. DSS was the name given to grades III and IV. A person must have a fever, hemorrhagic symptoms, thrombocytopenia, and evidence of plasma leakage to be diagnosed with DHF [10]. In addition to the symptoms seen in DHF patients, patients who develop DSS also show signs of shock, hypotension, a further narrowing of the pulse pressure, and a rise in HCT levels [11,12].

The adoption of new criteria in 2009 was prompted by the challenge of fulfilling the stringent requirements of DHF. Dengue without warning symptoms, Dengue with warning signs, and severe Dengue (SD) were the three categories under the revised criteria for symptomatic dengue. Abdominal discomfort and tenderness, continuous vomiting, mucosal bleeding, tiredness, restlessness, liver enlargement greater than 2 cm, and laboratory findings of elevated HCT value, together with a sharp drop in platelet count, are all warning signals. When severe bleeding, serious organ involvement, or severe plasma leakage resulting in shock or respiratory difficulty is present, SD is diagnosed [13]. The lady from our case had epigastric pain which is one of the warning signs of dengue. Pregnant women with sickle cell disease are a vulnerable population among whom dengue infection causes more fear in relation to the outcome of pregnancy. This is 10-fold among pregnant women with SCD and dengue [14]. Dengue fever during pregnancy may increase the risk of complications and cause dengue transmission from mother to child [15].

Among the various genotypes of sickle haemoglobin disease, sickle beta plus thalassemia (Sβ+Thal), sickle beta zero thalassemia (Sβ0thal), sickle cell anaemia with alpha thalassemia (SS αthal), and sickle cell anaemia with high foetal haemoglobin (SS+F) are included in sickle cell disease [2]. A homozygous mutation (haemoglobin S) is the cause of sickle cell anaemia, which manifests as chronic anaemia with excruciating episodes. The primary abnormality that causes these occurrences is compromised microcirculation due to erythrocyte sickling. These patients are susceptible to all maternal problems, including infections and hemolytic and thrombotic sickling crises. In patients with SCD who are pregnant, the presentation of dengue fever can be atypical and more severe. The co-existence of these two conditions can lead to a more complicated clinical picture. For instance, the vaso-occlusive crises commonly seen in pregnant women with SCD can be triggered or exacerbated by the febrile illness and inflammatory response associated with dengue infection. Additionally, the haemolysis in SCD pregnancies can be aggravated by the dengue virus, leading to severe anaemia and a higher likelihood of requiring blood transfusions. In this condition, there's a higher rate of morbidity and mortality among mothers and foetuses [16]. Patients with sickle cell disease usually have low concentrations of haemoglobin as seen in our case. The amount of haemolysis occurring can be gauged by the value of bilirubin. Urine culture rules out asymptomatic bacteriuria. 

As described by Koshy in their study sickle cell disease and pregnancy, pre pregnancy counselling along with regular assessment and prompt treatment with blood transfusions if necessary should be done [2]. 

The intersection of dengue fever and SCD pregnancy is particularly concerning due to the potential for exacerbated clinical outcomes. Mothers with SCD already face an increased risk of infections and complications, which the pathophysiology of dengue fever may further compound. Awareness and prompt management of potential complications, such as acute chest syndrome, multi-organ failure, and severe bleeding, are critical. These complications may require intensive care and multidisciplinary management. The prognosis of dengue fever in SCD pregnancies can be guarded, depending on the severity of the dengue infection and the underlying sickle cell disease. Early recognition and aggressive management of complications can improve outcomes. Research suggests that the interplay between the inflammatory response in dengue and the chronic inflammatory state in SCD may lead to more severe disease manifestations, thereby necessitating a high index of suspicion and proactive management during pregnancy.

## Conclusions

It is essential to prevent a pregnant woman with sickle cell disease and dengue from spiralling into a sickle cell crisis by active intervention. This case highlights the importance of early recognition and aggressive management of pregnant women's dengue fever in patients with sickle cell disease. The clinical signs and symptoms along with the laboratory results need to be carefully monitored to identify and manage the risk of severe haemolysis, vaso-occlusive crisis, hepatitis and bacterial co-infections. Multidisciplinary care involving haematologists, neonatologists and critical care teams is essential for optimising patient and improving outcomes as done in our case.
